# Preliminary phylogenetic insights into Japanese willows (*Salix*) using low-copy nuclear genes, with emphasis on endemic species

**DOI:** 10.1007/s10265-026-01728-x

**Published:** 2026-06-15

**Authors:** Satoshi Kikuchi, Suzuki Setsuko, Teruyoshi Nagamitsu, Wajiro Suzuki

**Affiliations:** 1 Hokkaido Research Center, Forestry and Forest Products Research Institute, 7 Hitsujigaoka, Toyohira, Sapporo, Hokkaido 062-8516 Japan; 2 Department of Forest Genetics, Forestry and Forest Products Research Institute, 1 Matsunosato, Tsukuba, Ibaraki 305-8687 Japan; 3 Tadami Beech Center, 2590 Machishita, Tadami, Tadami-machi, Fukushima 968-0421 Japan

**Keywords:** Chloroplast, Divergence time estimation, Japanese Archipelago, Low-copy nuclear genes, Phylogenetic networks, *Salix*

## Abstract

In this study, we aimed to reveal the phylogenetic relationships of *Salix* species in Japan, with particular emphasis on the speciation process of endemic species. We performed molecular phylogenetic analyses of all available native species using multilocus datasets of low-copy nuclear genes and chloroplast sequences. Although gene-tree incongruence and basal reticulation were evident—implying the need for future genome-scale analyses—our analyses still provided sufficient resolution to clarify the phylogenetic structure of the Japanese *Salix* flora and to offer meaningful insights into its diversification. The integrated analyses identified three major lineages within the Japanese subg. *Vetrix*, revealed the polyphyly of the subg. *Chamaetia,* and clarified the phylogenetic context of several key endemic species. For example, *S. hukaoana* of the monotypic section *Hukaoana* and *S. futura* were inferred to be relicts of ancient lineages that diverged during the Mid to Late Miocene, whereas the species of sect. *Hastatae* (i.e., *S. rupifraga*, *S. shiraii*, and *S. japonica*) were suggested to be neoendemics derived from *S. vulpina*. Moreover, intraspecific taxa such as *S. miyabeana* and *S. nakamurana* showed signs of hybridization, suggesting that interspecific hybridization and introgression may contribute to lineage differentiation.

**Supplementary Information:**

The online version contains supplementary material available at 10.1007/s10265-026-01728-x.

## Introduction

The genus *Salix* L. (Salicaceae) comprises approximately 400 tree species that are mainly distributed in the temperate, boreal, and Arctic regions of the Northern Hemisphere (Argus 1997). They are present in diverse ecological niches, including wetlands, riparian vegetation, uplands, and alpine/arctic tundra (Dickmann and Kuzovkina 2014; Newsholme 1992), and have diverse economic and ecological uses, including biomass production, ecological restoration, and various wood products (Pučka and Lazdiņa 2013).

A comprehensive classification of *Salix* has been attempted by many taxonomists; however, this has proven to be difficult because of the dimorphic sexual system, simple flowers, large phenotypic variations, frequent hybridization, and polyploidization of this genus (Argus 1997; Cronk et al. 2015). The current classification of *Salix* is based on several authoritative taxonomic opinions proposed for each region (Argus 1997; Dickmann and Kuzovkina 2014; Fang et al. 1999; Ohashi 2001; Skvortsov 1999).

Many molecular phylogenetic studies have been conducted to untangle the taxonomic and systematic complexity of willows (Acar et al. 2022; Azuma et al. 2000; Barkalov and Kozyrenko 2014a,b; Chen et al. 2010; Hardig et al. 2010; He et al. 2021; Lauron-Moreau et al. 2015; Sanderson et al. 2023; Wagner et al. 2018, 2021): early studies based on chloroplast and nuclear ribosomal genes revealed key evolutionary trends in the genus *Salix* and contributed to proposed taxonomic rearrangements at subgenus levels. In particular, evidence confirmed that *Salix* subgenera *Vetrix* Dumortier and *Chamaetia* (Dumortier) Nasarov formed a mixed clade, suggesting their recent diversification and the repeated evolution of dwarf arctic/alpine willows (subg. *Chamaetia*) (Acar et al. 2022; Azuma et al. 2000; Barkalov and Kozyrenko 2014a; Chen et al. 2010; Hardig et al. 2010; Lauron-Moreau et al. 2015; Sanderson et al. 2023). Recent advances in genomic sequencing have enabled the high-throughput phylogenetic analysis of willows, thereby revealing the complex evolutionary history of this clade at lower taxonomic levels, particularly within the *Vetrix–Chamaetia* complex (Chen et al. 2025; He et al. 2021; Ogutcen et al. 2024; Sanderson et al. 2023; Wagner et al. 2018, 2021).

*Salix* species in the Japanese Archipelago have been well described: The most updated classification of *Salix* by Ohashi (2001) classified the genus into six subgenera, namely, *Salix, Protitea*, *Pleuradenia*, *Chosenia*, *Chamaetia* and *Vetrix*, and describes 27 native species (Table 1), of which the majority (17) belong to subg. *Vetrix* (further classified into 9 sections), with three alpine dwarf willows included in the subg. *Chamaetia* (3 sections)*.* Although they apparently have links with continental East Asia, including Northeast China, the Korean Peninsula, Far Eastern Russia, and—in rare cases—Europe, they also show a moderate level of endemism (30%), harboring seven endemic species and two subspecies. However, phylogenetic research on *Salix* has not progressed in Japan since the earlier classical studies (Azuma et al. 2000), and the systematic origins of these species remain unclear.

**Table 1:** List of *Salix* species native to Japan and their taxonomic status (Ohashi 2001), along with the number of samples analyzed in this study. Ploidy levels are based on the previous reports including Suda (1964), Suda and Argus (1969) and Wagner et al. (2020)

Subgenus	Section	Species	Ploidy	N	Comments
*Pleuradenia* Kimura		*Salix cardiophylla* Trautv. & Mey	2X	3	
*Chosenia* (Nakai) H.Ohashi		*Salix arbutifolia* Pall	2X	1	
*Protitea* Kimura		*Salix chaenomeloides* Kimura	2X	4	
*Chamaetia* (Dumortier) Nasarov	*Herbella* Seringe	*Salix nummularia* Andersson	2X	2	
	*Myrtilloides* (Borrer) Andersson	*Salix fuscescens* Andersson	2X	3	
	*Glaucae* (Fries) Andersson	*Salix nakamurana* Koidz			
		subsp. *nakamurana*	unknown	2	Endemic to Japan
		subsp. *yezoalpina* (Koidz.) H.Ohashi	unknown	3	Endemic to Japan
		subsp. *kurilensis* (Koidz.) H.Ohashi	unknown	1	
*Salix* L	*Triandrae* Dumortier	*Salix triandra* L	2X	3	
	*Subalbae* Koidz	*Salix eriocarpa* Franch. & Sav	4X,5X	1	
		*Salix pierotii* Miq	4X	2	
		*Salix jessoensis* Seemen	4X,6X	5	Endemic to Japan
*Vetrix* Dumortier	*Hastatae* (Fries) A.Kerner	*Salix japonica* Thunb	2X	2	Endemic to Japan
		*Salix shiraii* Seemen	2X	1	Endemic to Japan
		*Salix rupifraga* Koidz	2X	2	
	*Sieboldianae* C.K.Schneid	*Salix sieboldiana* Blume	2X	4	Endemic to Japan
		*Salix reinii* Seemen	8X	2	
	*Helix* Dumortier	*Salix miyabeana* Seemen			
		subsp. *miyabeana*	4X	2	
		subsp. *gymnolepis* (H.Lév. et Vaniot) H.Ohashi et Yonek	4X	2	
		*Salix integra* Thunb	2X	2	
	*Incubaceae* A.Kerner	*Salix subopposita* Miq	2X	1	
	*Subviminales* C.K.Schneid	*Salix gracilistyla* Miq	2X	7	Including one sample from Korea
	*Hukaoana* Kimura	*Salix hukaoana* Kimura	unknown	15	Endemic to Japan
	*Daphnella* Seringe	*Salix rorida* Lacksch	2X	9	Including f. *pendula* Kimura (1) and f. *roridaeformis* (Nakai) Kimura ex H.Ohashi (1)
	*Viminella* Seringe	*Salix schwerinii* E. Wolf	2X	4	
		*Salix udensis* Trautv. & Mey	2X	13	
	*Cinerella* Seringe	*Salix taraikensis* Kimura	unknown	1	
		*Salix caprea* L	2X	11	Including samples from Korea (2) and European subspecies subsp. *coaetanea* (Hartm.) Hiitonen (1)
		*Salix futura* Seemen	3X	2	Endemic to Japan
		*Salix vulpina* Andersson	2X	4	

In the present study, we aimed to reveal the phylogenetic distinctiveness and affinity among Japanese *Salix* taxa. We particularly focus on several endemic taxa within the subg. *Vetrix–Chamaetia* complex and assess hypotheses concerning their systematic origins, as inferred from their distributional patterns and present taxonomic assignment. One example of such endemic species is *S. hukaoana* Kimura, which was first discovered in 1972 (Kimura 1973), and is currently known as a major component of the mountainous riparian forests of northern Honshu Island (Kikuchi and Suzuki 2010). It was once considered to be related to *Salix* sect. *Daphnella*, but was soon given a novel monotypic section *Hukaoana* on its unique morphological traits (Kimura 1973, 1974). We developed the following hypothesis of the hybrid origin of *S. hukaoana* (*S. gracilistyla* Miq. × *S. rorida*), based on its intermediate morphological traits and distribution: (1) connate stamens in male flowers shared with *S. gracilistyla* (and sect. *Helix*); (2) the yellow inner bark shared with sect. *Daphnella* (including *S. rorida*); (3) sympatric occurrence and hybrid formation with *S. gracilistyla*; and (4) parapatric distribution with *S. rorida*, sharing similar ecological niches as tall trees constituting upper mountainous riparian forests in northern Japan (Kikuchi and Suzuki 2010).

Another example comprises three local endemic species—*S. rupifraga* Koidz.*, S. shiraii* Seemen*,* and *S. japonica* Thunb. Their taxonomic assignment to *Salix* sect. *Hastatae* (Fries) A. Kerner indicates close phylogenetic relationships with exotic consectional species. However, their localized distributions to narrow volcanic areas (Ohashi and Yonekura 2006) may suggest recent speciation (e.g., Sciandrello et al. 2020). Furthermore, this study addressed some intraspecific taxa, including two subspecies of *S. miyabeana* Seemen and three of *S. nakamurana* Koidz., which are sometimes regarded as distinct species by some taxonomists.

Here we acquired multilocus sequences from low-copy nuclear genes (Sang 2002), and conducted a phylogenetic network analysis and divergence time estimation to reveal the divergence patterns and time scales of the focal species. Low copy genes are convenient and informative phylogenetic tools and are expected to serve as a preliminary insight into the phylogenetic origins of Japanese willows preceding future genomic research.

## Materials and methods

### Data collection

This study covered all willow species native to Japan (Table 1, Online Resource 1), along with 18 foreign willows and five (two native and three foreign) poplars (*Populus* L.) as outgroups. Leaf samples were collected from botanical gardens, herbarium specimens, and individuals in the field (Table 1). DNA was extracted from leaves using a DNeasy Plant Mini Kit (Qiagen, Maryland, USA) and diluted to a concentration of ~ 1 ng µL^−1^.

We obtained sequences from three chloroplast intergenic regions (*trnL*-*trnF*, *trnR*-*trnN*, and *atpB*-*rbcL*) and three low-copy nuclear (COS) genes (chloroplast-expressed glutamine synthetase (*ncpGS*), glucose-6-phosphate isomerase (*PGI*), and 6-phosphogluconate dehydrogenase (*6PG*)). Universal primers (Suyama et al. 2000; Taberlet et al. 1991; Terachi 1993) were used to amplify the chloroplast regions, whereas specific primers for the amplification of nuclear genes were designed to target the exonal regions (Table 2) using OLIGO version 6.65 (Molecular Biology Insights, Inc.). These primers were designed for the target sequence of *Populus trichocarpa* Torr. & Gray retrieved from the JGI PhycoCosm database (Grigoriev et al. 2021; https://phycocosm.jgi.doe.gov/phycocosm/home).

**Table 2:** List of the amplifying and reading primers developed for this study

	Gene	Type	Primer name	Sequence (5′-3′)	Location
*PGI*	glucose-6-phosphate isomerase	amplifying/reading primer (Forward)	Poptr_PGI + 2151	AAATGTAGATCCTATTGATGTTG	CDS(exon)
		amplifying/reading primer (Reverse)	Poptr_PGI − 2976	GCTGATCAATGCTTGATGCTCC	CDS(exon)
		internal primer (Reverse)	Poptr_PGI − 3442	TTGTTAGGATCAATGCCAAACT	CDS(exon)
*ncpGS*	glutamine synthetase leaf isozyme	amplifying/reading primer (Forward)	Poptr_ncpGS + 1490	GATGCACATTATAAGGCTTG	CDS(exon)
		amplifying/reading primer (Reverse)	Poptr_ncpGS − 2449	AATGTGTTCCTTATGGCGAAG	CDS(exon)
		internal reading primer (Reverse)	Poptr_ncpGS − 2252	GGTGTGGCATCCAGCACC	CDS(exon)
		internal reading primer (Forward) specific for subg. Vetrix–Chamaetia	Poptr_ncpGS + 1848	CAGTATCCTTGTCAAAGATTTG	intron
*6PG*	6-phosphogluconate dehydrogenase	amplifying/reading primer (Forward)	Poptr_6PG + 67	GCCCTTAATATCGCAGAG	CDS(exon)
		amplifying/reading primer (Reverse)	Poptr_6PG − 1195	TGGCAAGATCAGGATTCCTATCA	CDS(exon)

PCR was performed using a PerkinElmer 9700 Thermocycler in 10 µL reaction mixtures. These consisted of ~ 0.5 ng template DNA, 20 mM Tris–HCl (pH 8.4), 50 mM KCl, 2.0 mM MgCl_2_, 0.2 mM of each dNTP, 0.15 µm of each primer and 0.25 U *Taq* polymerase. The PCR conditions were as follows: 94 °C for 3 min, followed by 35–40 cycles of 94 °C for 1 min, 55 °C for 1 min and 72 °C for 2 min, with a final extension of 72 °C for 5 min. PCR products were then purified (ExoSAP-IT, Amersham Biosciences) before being subjected to cycle sequencing using an ABI Big Dye™ Terminator Cycle Sequencing Kit version 3.1 (Applied Biosystems, Foster City, USA), and finally analyzed on an ABI 3100 automated sequencer (Applied Biosystems). Sequences were read in both directions using forward and reverse amplification primers; if needed, we also used internal sequencing primers designed for this study (Table 2).

Sequence editing and assembly were performed using the CodonCode Aligner version 3.7.1 (CodonCode Corporation) to generate consensus sequences. Heterozygous substitutions in the nuclear genes were coded using IUPAC ambiguity codes. Sequences with multiple heterozygous indels were not successfully assembled and excluded from further analyses. All assembled sequences are registered in the DNA Data Bank of Japan (Online Resource 1). Multiple sequence alignments were performed using the ClustalW2 algorithm, as implemented in the SeaView alignment editor (Gouy et al. 2010). Nuclear sequences were then phased into haplotypes using the PHASE algorithm as implemented in DnaSP version 6 (Librado and Rozas 2009) and used to compute haplotype diversity (*Hd*), nucleotide diversity (*Pi*), and Tajima’s *D* (Tajima 1989) to test the neutral evolution hypothesis.

### Phylogenetic reconstruction

Gene trees were constructed for four loci—one chloroplast region and three nuclear genes—based on the successfully assembled data (Online Resource 1). For the three nuclear loci, both unphased and phased datasets were prepared and analyzed. The sequences were collapsed into unique haplotypes/genotypes using DnaSP to generate reduced datasets. *Populus* species, and if not available, *S. chaenomeloides* Kimura, were used as the outgroup.

Phylogenetic reconstruction was performed using maximum likelihood (ML) and Bayesian inference (BI) methods. Prior to analysis, exonic regions of the nuclear *ncpGS* and *PGI* genes were identified via the JGI PhycoCosm database and removed, whereas the *6PG* gene, which is entirely exonic, was analyzed without modification. ML trees were constructed using RAxML version 8.2.10 (Stamatakis 2014) with a raxmlGUI 2 (Edler et al. 2021) graphical interface, and the bootstrap confidence values of the nodes were evaluated by generating one thousand bootstrap replicates. The BI method was executed using MrBayes version 3.2.7 (Ronquist et al. 2012). Two independent runs containing four Markov chain Monte Carlo chains (one hot and three cold) were performed until the average standard deviation of the split frequencies fell below 0.01. Trees were saved every 500 generations and the first 10% were discarded as burn-in.

The optimal substitution models were selected using jModelTest version 2.1.10 (Posada 2008) for the chloroplast and phased *ncpGS* and *PGI* gene sequences, and the alternative supported models were employed in MrBayes and RAxML analyses. For the other data sets (including the unphased nuclear genes and the phased sequences of the *6PG* gene), we ran a reversible-jump MCMC (rjMCMC) implemented in MrBayes and applied a GTR + Γ + I model in RAxML.

Moreover, evolutionary relationships within the genus *Salix* were visualized using phylogenetic network analysis. A combined data matrix was generated for the 68 samples associated with a complete dataset by concatenating the unphased sequences of all genes, including both exonic and intronic sequences. A phylogenetic network was constructed using the NeighborNet algorithm based on the uncorrected P distance, as implemented in SplitsTree 6.6.1 (Huson and Bryant 2006). Split support values were computed using 1,000 bootstrap replicates.

Additionally, phylogenetic network analysis was performed to test for the hybrid origin of species and for the *ncpGS* and *PGI* genes separately, as the focal species suspected of a hybrid had an incomplete dataset. This analysis incorporated partially assembled sequences of the focal species, that is, the *ncpGS* sequence from *S. nakamurana* subsp. *nakamurana* and the *PGI* sequence of *S. miyabeana* subsp. *miyabeana*.

### Divergence time estimation

The divergence time was estimated using a Bayesian inference method implemented in BEAST version 2.6.7 (Drummond and Rambaut 2007). We performed species tree analysis using StarBEAST3 and estimated posterior mean values and 95% highest posterior density (HPD) intervals of divergence time for all nodes. We used a Yule speciation prior and applied a species tree relaxed clock model and HKY substitution model.

Molecular dating was calibrated using fossil records as follows (Wu et al. 2015). The first calibration point was set at 48 Ma (normal distribution, SD = 0.3) for the root node of Salicaceae sensu* strict.* This was based on an approximately 48-million-year-old fossil from the early Eocene in North America (*“Populus tidwellii”*), which most likely represents the stem lineage leading to *Populus* and *Salix* (Manchester et al. 2006). The second calibration was set at 23 Ma (normal distribution, SD = 0.3) for the root node of the *Vetrix–Chamaetia* clade based on the earliest reliable *Salix* fossils from Late Oligocene deposits (23 Ma) in Alaska, which were found to be affiliated with subg. *Vetrix* (Wolfe 1987). This method of calibration differed from that used in previous studies (He et al. 2021; Wu et al. 2015), which used the age of the earliest *“Vetrix”* fossils to calibrate the nodes for the divergence between *Vetrix* and *Chamaetia*. Instead, we performed fossil calibration of the most recent common ancestor of the *Vetrix–Chamaetia* clade, since subg. *Chamaetia* was found to be polyphyletic. We ran the MCMC chains for 100,000,000 generations, sampling every 50,000th generation, using Tracer to ensure that the runs converged and had ESS values of > 200. Consensus trees were calculated after discarding the first 10% of trees as burn-in. Only the highly variable intronic (unphased) sequences of the *ncpGS* and *PGI* genes were subjected to this analysis because preliminary multispecies coalescent runs that incorporated all four loci failed to converge despite extended MCMC chains.

## Results

Sequences containing multiple heterozygous indels were often detected in nuclear *ncpGS* and *PGI* genes, particularly in the polyploid *Populus* and *Salix* subg. *Salix* species (Table 1). After omitting these, we obtained 711 assembled sequences with a total of 5,428 aligned base pairs (bp) from the chloroplasts and three nucleotide genes (GenBank accession numbers LC757833-758526 and LC859069-859110, Online Resource 1). Based on the phased nuclear and haplotypic chloroplast sequences, the loci differed markedly in their levels of polymorphism, with parsimony-informative sites ranging from 94 in the chloroplast regions to 181 in *PGI* (*ncpGS*: 162) and nucleotide diversity (π) ranging from 0.00517 (chloroplast) to 0.02752 (*ncpGS*). These patterns indicate that the nuclear intronic loci (*ncpGS* and *PGI*) contain substantially greater phylogenetically informative variation than the exon-dominated *6PG* gene or the chloroplast regions. Summary statistics are presented in Table 3. The best-fit models of nucleotide substitutions for nuclear intronic sequences in the *ncpGS* and *PGI* genes and the chloroplast *atpB-rbcL*, *trnR-trnN*, and *trnL-trnF* intergenic regions were the TPM2uf + G, HKY + G, TPM3uf, F81 + I, and HKY + G models, respectively.

**Table 3:** Summary of sequence characteristics and genetic diversity statistics for the four loci (6*PG*, *ncpGS*, *PGI*, and chloroplast sequences)

Measure	6*PG*	*ncpGS*	*PGI*	Chloroplast (concatenated)
Number of phased/haplotypic sequences	266	188	160	134
Aligned sequence length	957	721	905	2724
Variable sites	143	173	199	121
Parsimony informative sites	115	162	181	94
π: nucleotide diversity (per site)	0.01017	0.02752	0.02285	0.00517
*Hd*: haplotype (gene) diversity	0.9387	0.962	0.955	0.894
Tajima's *D*	− 1.94209	− 1.56752	− 1.5044	− 1.51405
Statistical significance	*p* < 0.05	0.10 > *p* > 0.05	*p* > 0.10	*p* > 0.10

Values shown include the number of phased/haplotypic sequences, aligned sequence length, numbers of variable and parsimony-informative sites, nucleotide diversity (π), haplotype diversity (*Hd)*, and Tajima’s *D* with associated significance levels

### Gene trees

**Fig. 1:**
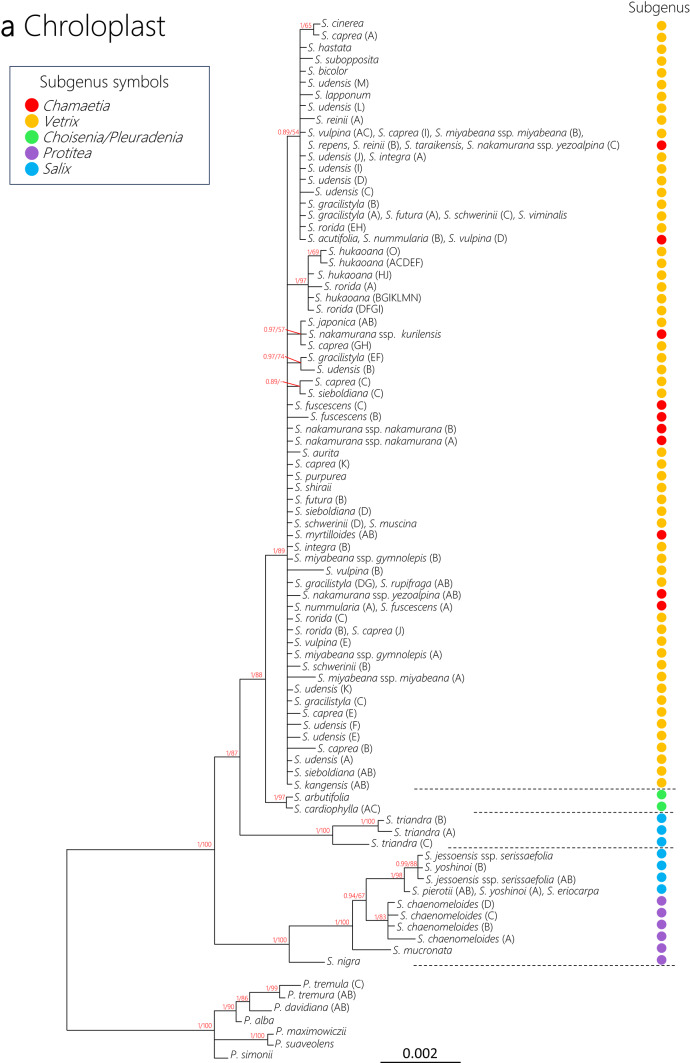

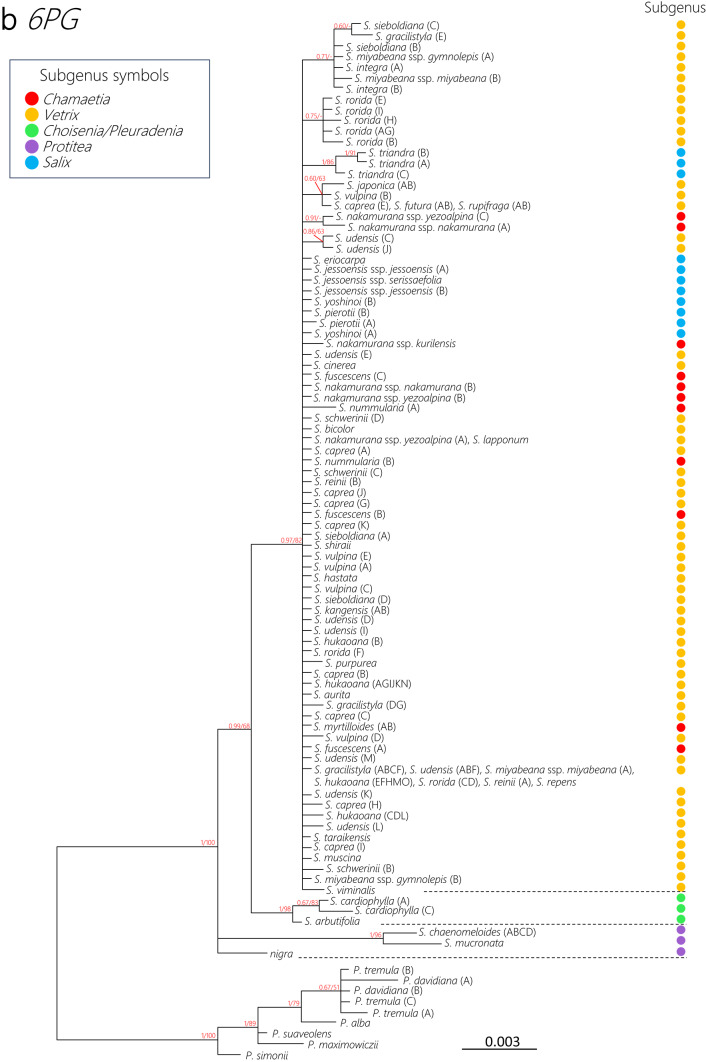

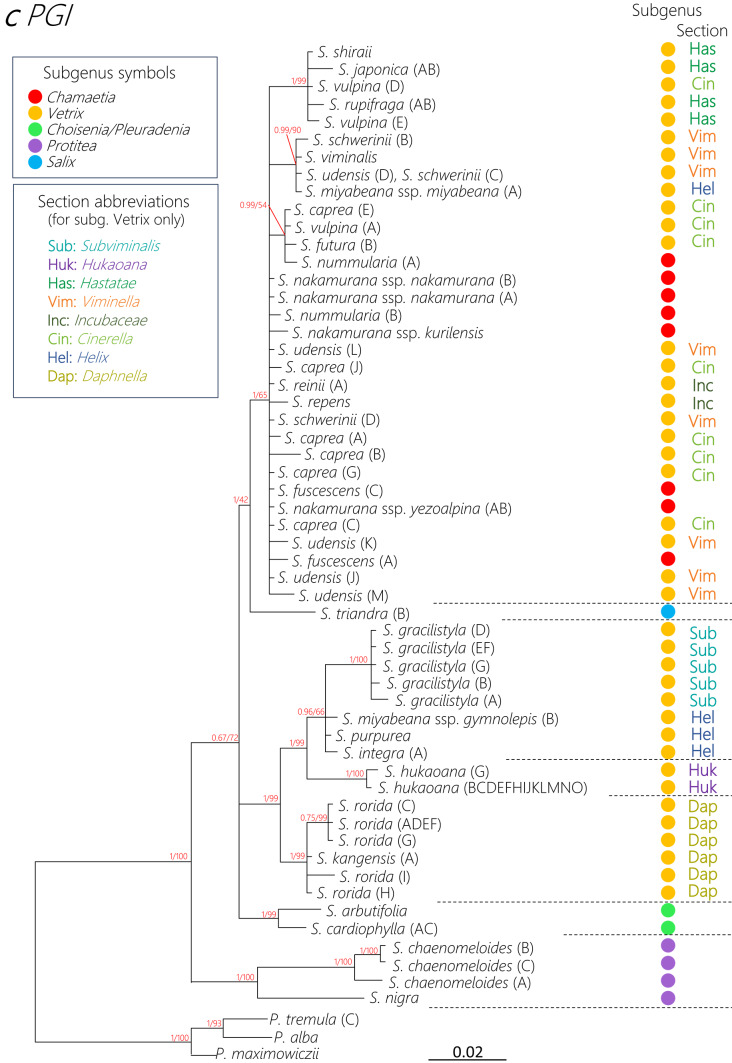

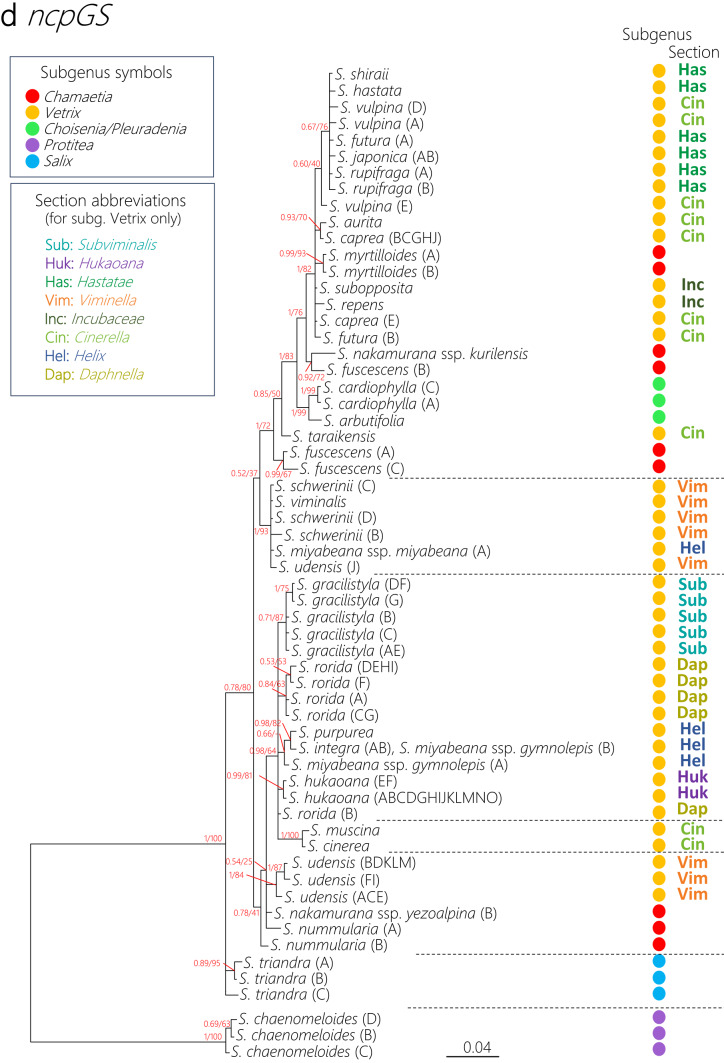
Phylogenetic trees (gene trees) based on **a** chloroplast, **b** nuclear 6*PG*, **c** *PGI*, and **d** *ncpGS* gene sequences were reconstructed using the Bayesian inference (BI) method. Sequences were collapsed into unique haplotypes/genotypes using DnaSP to form reduced datasets. The numbers above the branches represent Bayesian posterior probability and MP bootstrap values. “– “ indicates that the node was not supported in MP analysis

Figure 1a-d shows Bayesian trees based on the chloroplast sequences and unphased sequences of the nuclear genes, with the bootstrap values of ML indicated. Those for the phased nuclear sequences are provided in Online Resource 2a-c. The data used to construct each gene tree are listed in Online Resource 1. The tree topologies obtained using the Bayesian and ML methods were mostly congruent, except for the *6PG* gene, for which only the major clades were supported by both methods.

The chloroplast phylogeny (Fig. 1a) indicates two diverging clades in *Salix*. One comprises subg. *Salix* (except *S. triandra* L.) and *Salix* subg. *Protitea* Kimura, and the other includes subg. *Vetrix–Chamaetia*, with *S. triandra* as the first diverging clade, followed by *S. arbutifolia* Pall (*Salix* subg. *Chosenia* (Nakai) H.Ohashi), and *S. cardiophylla* Trautv. & Mey. (*Salix* subg. *Pleuradenia* Kimura). The phylogenetic relationships at lower taxonomic levels were not resolved, except that *S. hukaoana* was located within a single lineage with some *S. rorida* Lacksch. specimens.

The exonic sequences of 6*PG* also provided poorly resolved phylogenies (Fig. 1b and Online Resource 2a). Unphased data confirmed the early divergence of subg. *Protitea* and the subgenera *Chosenia* and *Pleuradenia*. (Fig. 1b). In contrast, gene genealogy based on phased data identified one allele from the subg. *Salix* (except for *S. triandra*) within the early diverging clades (Online Resource 2a). The phylogenetic relationships within the *Vetrix–Chamaetia* clade were not resolved, except that *S. triandra* was shown to be monophyletic.

Intronic sequences in nuclear COS genes (i.e., *ncpGS* and *PGI*) provided highly resolved phylogenetic trees, although the low success of sequence assembly reduced the number of analyzed taxa, particularly for polyploid species of sect. *Sieboldianae* C. K. Schneid. (*S. sieboldiana* Blume and *S. reinii* Seemen) along with subg. *Salix* (i.e., *S. eriocarpa* Franch. & Sav., and *S. jessoensis* Seemen; Table 1). The major clade comprising subg. *Vetrix–Chamaetia* together with *S. arbutifolia, S. cardiophylla*, and *S. triandra* was maintained in both trees, although *S. arbutifolia* and *S. cardiophylla* were nested within the *Vetrix–Chamaetia* clade in the *ncpGS* gene tree (Fig. 1c-d and Online Resource 2b-c). Although a common pattern was observed between the two nuclear loci regarding the internal structure of subg. *Vetrix–Chamaetia*, in which one lineage consistently grouped in *Salix* sects. *Hukaoana* Kimura, *Daphnella* Seringe, *Subviminales* C. K. Schneid., and *Helix* Dumortier, the gene trees exhibited substantial topological incongruence. Our analyses also indicated that *S. miyabeana* (sect. *Helix*) is polyphyletic, with subsp. *miyabeana* located distantly from subsp. *gymnolepis* (H. Lév. et Vaniot) H. Ohashi et Yonek. and is instead closely related with *S. schwerinii* E. Wolf (sect. *Viminella* Seringe).

### Phylogenetic network

**Fig. 2:**
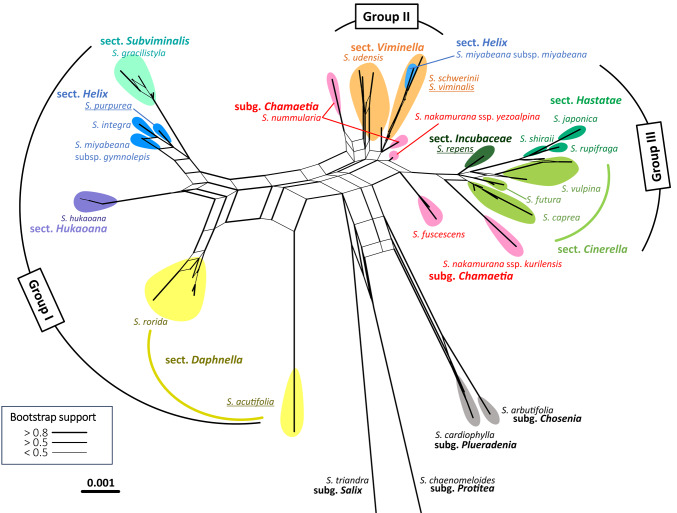
NeighborNet network of *Salix*, focusing on Japanese native species of the subg. *Vetrix–Chamaetia* complex. The network was constructed using uncorrected P distances based on concatenated sequences of chloroplast and three nuclear genes. Splits with bootstrap support > 50% and > 80% were shown using increasingly thicker line weights

NeighborNet analysis involved 67 samples from 24 species for which successfully assembled sequences of all genes were available, and therefore lacked some native species (i.e., *S. subopposita* Miq., *S. sieboldiana*, *S. reinii*, and *S. taraikensis* Kimura). Nevertheless, the phylogenetic network (Fig. 2) effectively resolved the species-level relationships within subg. *Vetrix–Chamaetia*. It represented three major lineage groups (Groups I-III) in which the Japanese species of subg. *Vetrix* evolved into, showing some basal reticulation. The first group (Group I) comprised sects. *Subviminales*, *Daphnella*, *Helix*, and *Hukaoana*. Within this group, *S. hukaoana* (sect. *Hukaoana*) showed distinct divergence without evidence of recent reticulation events, while the species of sect. *Helix* were placed near a peripheral reticulation.

The second group (Group II) almost exclusively consisted of sect. *Viminella* with the exception of *S. miyabeana* subsp. *miyabeana*, positioned distantly from subsp. *gymnolepis*. This group further divides into two sublineages (*S. udensis* and the others), whose terminal branches are connected via peripheral reticulations.

The third group (Group III) included *Salix* sects. *Cinerella* Seringe, *Hastatae* and *Incubaceae* A.Kerner. Notably, sects. *Cinerella* and *Hastatae* showed progenitor-derivative relationships, where the species of sect. *Hastatae* (i.e., *S. japonica*, *S. rupifraga*, and *S. shiraii*, all endemic species) descended from *S. vulpina* Andersson. In contrast, *S. futura* Seemen, another endemic species, diverged from the roots of this group, sharing a reticulation with *S. vulpina*.

In contrast, the alpine dwarf willows (subg. *Chamaetia*) branched from the bases of the phylogenetic network, with *S. nummularia* Andersson at the base of Group II, *S. nakamurana* subsp. *nakamurana* at the base of Group III, while the others located at the intermediate positions.

The gene phylogenetic networks (Online Resource 3a, b) were less resolved but helped to detect the occurrence of hybridization. While the phased *PGI* sequences of *S. miyabeana* subsp. *miyabeana* (A) were positioned close to *those of S. schwerinii*, those of the sample (B) fell into separate positions, one close to the sect. *Viminella* and another close to subsp. *gymnolepis.* At the 14 heterozygous sites recognized from this sample, one sequence variant matched *S. miyabeana* subsp. *gilgiana*, whereas the other matched multiple potential species including *S. schwerinii*, *S. vulpina*, *S. udensis*, *S. caprea*, *S. nummularia*, *S. nakamurana*, and *S. reinii* (Online Resource 4). The phased *ncpGS* sequences of S. *nakamurana* subsp. *nakamurana* (B) also showed signs of hybridization, with one grouped with subsp. *kurilensis* and the other falling close to sect. *Viminella*. At the 13 heterozygous sites, one sequence variant matched *S. nakamurana* subsp. *kurilensis*, whereas the other matched subsp. *yezoalpina*, (Online Resource 4).

### Divergence time estimation

**Fig. 3:**
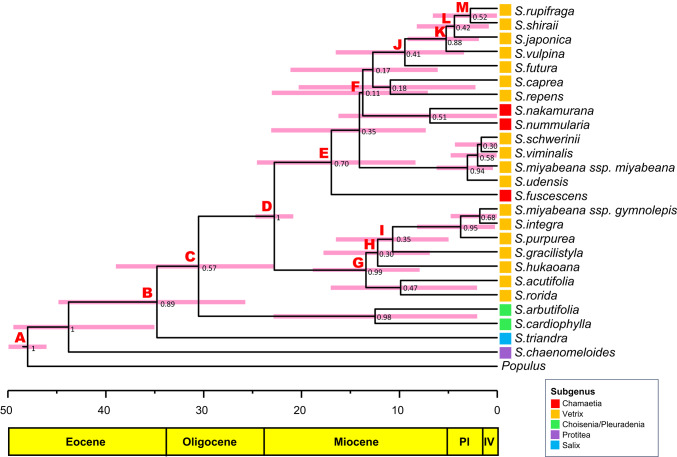
Bayesian divergence time estimates of Japanese native *Salix* species in millions of years ago (Mya), based on nuclear intronic sequences of the *ncpGS* and *PGI* genes. Pink bars at each node indicate the 95% highest posterior density (HPD) interval of divergence time. Mean divergence time and 95% HPD for lettered nodes (A–N) are listed in Table 4. Node support is given by Bayesian posterior probabilities shown to the left of each node. Abbreviation of the periods: Pl—Pliocene, IV—Quaternary

The estimated divergence times at the representative nodes are presented in Fig. 3 and summarized in Table 4. They suggest that the *Helix/Daphnella/Hukaoana/Subviminales* lineage group was diversified at 13.4 Ma, followed by the divergence of *S. hukaoana* at 12.2 Ma. The divergence of *S. futura* was estimated to have occurred approximately 9.4 Ma, whereas the endemic species group (classified as sect. *Hastatae*) was estimated to have diverged 5.2 Ma, with diversification starting around 4.4 Ma and speciation of the alpine-adapted species *S. rupifraga* at 2.7 Ma.

**Table 4:** Mean divergence time estimates (Mya) of representative nodes (i.e., lettered nodes in Fig. 3) for Japanese native *Salix* species and lineage based on nuclear intronic sequences of the *ncpGS* and *PGI* genes

Node	Event	Mean divergence time (95% HPD) (Ma)
A	Root node of Salicaceae*	48.03 (46.09–49.99)
B	Divergence of *S. triandra*	34.80 (25.76 –44.87)
C	Divergence of *S. arbutifolia/cardiophylla* (Divergence of *Vetrix–Chamaetia*)	30.54 (22.71–39.00)
D	Crown age of subg. *Vetrix–Chamaetia* *	22.79 (20.85–24.72)
E	Divergence of subg. *Chamaetia* (*S. fuscescens*)	16.96 (8.34–24.57)
F	Divergence of subg. *Chamaetia* (*S. nakamurana*/*nummularia*)	13.74 (7.08–23.04)
G	Crown age of the *Daphnella*–*Subviminalis*–*Helix*–*Hukaoana* clade	13.42 (7.92–18.85)
H	Divergence of *S. hukaoana*	12.21 (6.88–17.76)
I	Divergence between sects. *Subviminalis* and* Helix*	10.68 (4.96–16.49)
J	Divergence of *S. futura*	9.43 (3.37–16.50)
K	Divergence of Fossa-Magna element (endemic species of subg. *Hastatae*)	5.21 (1.86–9.16)
L	Crown age of endemic species of subg. *Hastatae*	4.36 (0.84–8.21)
M	Divergence of* S. rupifraga*	2.72 (0–6.57)

^*^Calibrated nodes

The 95% highest posterior density (HPD) interval is shown in parentheses

## Discussion

Recently, low-copy nuclear genes have become more widely used (Sang 2002; Zimmer and Wen 2013) as tools for robust phylogenetic reconstruction and taxonomic resolution. Although they have some disadvantages such as difficulties in isolating orthologous alleles and potential discordance due to incomplete lineage sorting and interspecific hybridization (Sang 2002; Small et al. 2004), phylogenetic inference based on multi-locus nuclear genes has proven to be a robust tool for addressing evolutionary relationships (Huson and Scornavacca 2011).

In our study, several sources of phylogenetic uncertainty were evident. Polyploid species, particularly those in subg. *Salix* (except *S. triandra*), often failed to assemble successfully or yielded divergent phased sequences (Fig. 1b; Online Resource 1a), suggesting the presence of multiple divergent gene copies. Such assembly difficulties in subg. *Salix* may reflect allopolyploid origins derived from inter-lineage hybridization within this clade, as recent studies on sex-determining regions have demonstrated evidence of reticulate evolution in *Salix* (Gulyaev et al. 2022; He et al. 2024; Xue et al. 2024); the locus-specific phylogenetic instability observed in our *6PG* analyses may be consistent with this complex genomic history. None of the individual loci provided sufficient resolution at species or sectional levels: the chloroplast markers and the exonic *6PG* gene exhibited low variability and did not resolve relationships within subg. *Vetrix–Chamaetia* (Fig. 1a, b; Online Resource 2a), whereas the intronic regions of *ncpGS* and *PGI* were more informative but still generated topological incongruence among gene trees (Fig. 1c, d; Online Resource 2b, c). In line with these patterns, the NeighborNet analysis revealed basal reticulation among the major lineages of the *Vetrix–Chamaetia* complex (Fig. 2), indicating that early diversification in *Salix* involved reticulate evolutionary processes that cannot be fully resolved using a limited number of loci likely reflecting incomplete lineage sorting and historical hybridization/introgression (Degnan and Rosenberg 2009).

Collectively, these findings underscore the limitations of single- or few-locus datasets for reconstructing deep relationships in *Salix* and highlight the need for future genome-scale analyses to disentangle gene discordance, detect ancient hybridization, and clarify lineage diversification among Japanese *Salix* species. Nevertheless, analysis based on the combined dataset—particularly the NeighborNet network—provided a certain level of phylogenetic resolution, most notably within subg. *Vetrix–Chamaetia* (Fig. 2) and yielded valuable insights into the systematic origins of key endemic species, as discussed below.

In parallel with the network analysis, divergence-time estimation using BEAST allowed us to place these phylogenetic patterns within a temporal framework. Previous studies have estimated divergence times in *Salix* (He et al. 2021; Marinček et al. 2024; Sanderson et al. 2023; Wu et al. 2015), although only a few have incorporated multiple fossil calibration points. By adopting a comparable calibration strategy, our estimates for major nodes (e.g., nodes B, C, F, and I and the divergence between *S. arbutifolia* and *S. cardiophylla*; Fig. 3) closely match those of earlier studies, thereby supporting the robustness of our dating results.

### Ancient origin of *S. hukaoana* and *S. futura*

Previous studies proposed that sects. *Helix, Daphnella*, and *Subviminales* have a high degree of relatedness (He et al. 2021; Wagner et al. 2018, 2021). We observed that *S. hukaoana* (sect. *Hukaoana*) belongs to this group (Group I). This group harbors morphologically diverse species, ranging from shrubs to tall trees, with lanceolate to elliptical leaves and opposite to alternate leaves. Although no obvious synapomorphies were observed, several traits were partially shared. This lineage likely emerged during the early evolutionary stage of subg. *Vetrix–Chamaetia* and diverged into variable sections by the Middle Miocene (Table 3). This finding is congruent with fossil records from the late Middle Miocene in Japan, which are thought to reflect extant species from sects. *Helix* and *Subviminales* (Narita et al. 2020).

Within this group, *S. hukaoana* was distinctly diverged from the other sections without recent reticulations, which disconfirms the recent hybrid-speciation hypothesis of *S. hukaoana* based on its intermediate traits and distribution (see Introduction). Therefore, our results indicate that the traits of *S. hukaoana* are not a by-product of recent hybrid speciation, but rather a combination of apomorphic and plesiomorphic characters (which may have stemmed from ancient reticulate evolution). Moreover, there still remains a possibility of secondary hybridization and introgression between *S. hukaoana* and *S. rorida*, as indicated by the presence of shared haplotypes/genotypes in the chloroplast and *PGI* genes (Fig. 1a; Online Resource 2b). The divergence time of *S. hukaoana* from other species examined in this study is estimated to be in the late Middle Miocene (ca. 12 Ma). Given that its closest extant relative known to date is S. baileyi C.K. Schneider, a shrub endemic to central and eastern China, *S. hukaoana* was regarded as a relict of ancient origin.

The other endemic species of ancient origin was *S. futura* (Group III, further discussed below), which was estimated to have split from the *S. vulpina* lineage a bit later (9 Ma) in the Late Miocene. These periods are characterized by a global cooling trend after the Middle Miocene Climatic Optimum (Pavlyutkin et al. 2016), and the megafossil flora in North Japan shows a dominance of *Fagus* species, suggesting an expansion of beech forests around that time. *S. futura* may be a relic lineage of early cool-temperate flora in Japan, which persisted despite the presence of derived species.

### Evidence of interspecific hybridization in *S. miyabeana* subsp. *miyabeana*

Subg. *Vetrix* of Group II was composed of narrow-leaved riparian species, and almost exclusively comprised of the sect. *Viminella,* which is a phylogenetic group well supported by the recent genomic studies (He et al. 2021; Wagner et al. 2018, 2020), with the exception of *S. miyabeana* subsp*. miyabeana* (sect. *Helix*).

*S. miyabeana* subsp. *miyabeana* is the type subspecies, distinguished from subsp. *gymnolepis* only by subtle morphological characters, including more elongated leaves, shorter styles, and nearly sessile ovary stipes. In addition, although subsp. *gymnolepis* typically bears a single connate stamen in each male flower, subsp. *miyabeana* occasionally produces male flowers with two stamens within the same inflorescence*.* In our study, the only sample with a full data set was shown to be distantly related to subsp. *gymnolepis* (Group I) and the most closely related to *S. schwerinii*, whereas another individual with an incomplete data set (sample B) showed evidence of hybrid origin (Online Resources 3a, 4).

*S. miyabeana* subsp. *miyabeana* is distributed more northerly (i.e., Hokkaido Island) than subsp. *gymnolepis* (i.e., the southern tip of Hokkaido to Honshu) and has a greater opportunity to grow sympatrically with *S. schwerinii* (northern Japan). These results suggested that *S. miyabeana* subsp*. miyabeana* was, at least, not phylogenetically homogeneous with subsp. *gymnolepis* and was likely subject to some degree of genetic introgression from *S. schwerinii*. We can hypothesize that intersectional hybridization with *S. schwerinii* has driven subspecies differentiation in *S. miyabeana*, however, clarifying the details of hybridization status—including the extent of hybridization/introgression and the occurrence of polyploidization in hybrids—will require examining a larger sample size and more extensive genetic data in future studies.

### Radiative speciation of local endemic species of “sect. *Hastatae*”

Subg. *Vetrix* in Group III comprises the round-leaved hillside willows of sects. *Cinerella* and *Hastatae*. Uncovered by the NeighborNet analysis, the placement of *S. taraikensis* (sect. *Cinerella*) remains unclear. Available evidence from the *PGI* tree suggests a close but distinct relationship with other *Cinerella* species (Fig. 1c; Online Resource 2c). This is noteworthy, as previous studies have identified early divergence in *S. starkeana* Willd. (Wagner et al. 2020, 2021)*,* which was classified together with *S. taraikensis* as the subsect. *Substriatae* Goerz by Eurasian taxonomists (Skvortsov 1999).

Group III represents an ancestor–descendant relationship between *S. vulpina* (subg. *Cinerella*) and the Japanese species of sect. *Hastatae*, with the latter being a derived monophyletic lineage that diverged from *S. vulpina* around 5.2 Ma and subsequently diversified at 4.4–2.7 Ma into three species (Fig. 3; Table 4). Previous studies (Marinček et al. 2024; Wagner et al. 2021) have suggested that sect. *Hastatae* is polyphyletic, and no previous study has documented its members being nested within sect. *Cinerella* as found in our study. These findings imply that the Japanese members of sect. *Hastatae* at least require taxonomic reevaluation.

More notably, our results may provide significant insight into the plant speciation process within the Japanese Archipelago. These three willow species are local endemics belonging to the so-called “Fossa Magna element,” a biogeographic group restricted to the Fuji Volcanic Zone, extending from the Izu Islands to central Honshu. The endemism of the Fossa Magna element is generally interpreted as having evolved through adaptation to volcanic environments (Takahashi 1971), which were created by the emergence of volcanic islands and repeated collisions with central Honshu since the Miocene (Maruyama et al. 1997; Takagi et al. 1993). Consistent with this scenario, the estimated divergence times of these endemic willows coincide with major volcanic episodes in this region and closely match that of *Rubus trifidus* Thunb. (6.9 Ma), another representative member of the Fossa Magna element (Kikuchi et al. 2022). Moreover, these willows exhibit striking ecological and morphological diversification—from hillside shrubs to alpine dwarf forms (Ohashi and Yonekura 2006)—suggesting that they have undergone adaptive radiation within this dynamic geological setting. Taken together, these findings may highlight volcanic and tectonic activity as key drivers of plant speciation in the Japanese Archipelago.

However, this scenario warrants cautious interpretation, as neither the present study nor previous investigations have yet produced a comprehensive phylogenetic framework for *Salix*. Given the substantial number of species that remain unexamined, it is possible that unsampled lineages may have played a role in the origin of the Japanese species of sect. *Hastatae*.

### Polyphyly and ancient divergence of subg. *Chamaetia*

The findings on subg. *Chamaetia* species in Japan obtained in this study—such as the polyphyly of this subgenus and the placement of *S. nummularia* and *S. fuscescens* at the basal position near sect. *Viminella* and near sect. *Cinerella*, respectively—are consistent with the results of previous studies (Lauron-Moreau et al. 2015; Marinček et al. 2024; Wagner et al. 2018, 2021).

In contrast, the phylogenetic position of *S. nakamurana* does not allow a clear interpretation. Our results showed distinct divergence between subsp. *kurilensis* and subsp. *yezoalpina* (Fig. 3), and the nuclear genes of subsp. *nakamurana* exhibit heterozygosity for divergent alleles corresponding to both lineages (Online Resources 3b, 4), a pattern consistent with hybridization between the two subspecies. Nevertheless, our analyses clearly demonstrate that the nuclear genes of *S. nakamurana* harbor highly divergent alleles within the species. The presence of highly divergent alleles within the species may indicate polyphyly of S. *nakamurana*, subspecific lineage divergence driven by geographic isolation, incomplete lineage sorting of ancestral alleles, or the influence of interspecific hybridization. Future genomic analyses will be required to evaluate these alternative scenarios.

Finally, our divergence-time estimates suggest that the origin and diversification of Japanese *Chamaetia* species—including *S. nakamurana*, *S. nummularia*, and *S. fuscescens*—date back to the Miocene (Fig. 3; Table 4). This timing corresponds to major climatic transitions, beginning with the warm Middle Miocene followed by global cooling and Antarctic ice sheet expansion (Herbert et al. 2016), and agrees with fossil evidence (Wolfe 1987) as well as recent estimates indicating that the radiation of shrub willows began in the Miocene (Marinček et al. 2024).

## Conclusion

Despite the methodological limitations of Sanger sequencing, phylogenetic analyses using nuclear COS genes and chloroplast sequences in this study provide significant insights into the evolutionary relationships and timescale of diversification of *Salix* species in Japan. In particular, the results clarified the origins of several endemic taxa through a range of speciation processes, identifying ancient relicts (*S. hukaoana* and *S. futura*) and recently derived neoendemics (*S. japonica*, *S. shiraii* and *S. rupifraga*), and possible cases of lineage differentiation influenced by interspecific/intraspecific hybridization (*S. miyabeana* subsp. *miyabeana* and *S. nakamurana* subsp. *nakamurana*). These findings underscore the complexity of evolutionary dynamics within the Japanese willow flora, highlighting the need for future genome-scale studies to test these hypotheses and clarify the speciation mechanisms of Japanese *Salix*.

## Supplementary Information

Below is the link to the electronic supplementary material.

Supplementary Material 1

Supplementary Material 2

Supplementary Material 3

Supplementary Material 4

Supplementary Material 5
