# Supplementary material for: Preliminary phylogenetic insights into Japanese willows (Salix) using low-copy nuclear genes, with emphasis on endemic species
Source: J Plant Res. 2026 Jun 15;139(4):575–91. doi: 10.1007/s10265-026-01728-x (PMC13332978; doi:10.1007/s10265-026-01728-x)
Supplement: Supplementary file 2 — Supplementary Material 2 [file 10265_2026_1728_MOESM2_ESM.pdf]

Title: Preliminary phylogenetic insights into Japanese willows (*Salix*) using low-copy nuclear genes, with emphasis on endemic species
Journal: Journal of Plant Research
Authors: Satoshi Kikuchi, Suzuki Setsuko, Teruyoshi Nagamitsu, Wajiro Suzuki
Affiliation: Hokkaido Research Center, Forestry and Forest Products Research Institute, Japan
Corresponding author: Satoshi Kikuchi
Email: kikuchi_satoshi450@ffpri.go.jp

Online Resource 2

Phylogenetic trees (gene trees) based on the phased sequences of (**a**) nuclear 6*PG*, (**b**) *PGI*, and (**c**) *ncpGS* genes as reconstructed using the Bayesian inference (BI) method. Sequences were collapsed into unique haplotypes using DnaSP to form reduced datasets. The numbers above the branches represent Bayesian posterior probability and MP bootstrap values. "–" indicates that the node was not supported in MP analysis.

a *6PG*

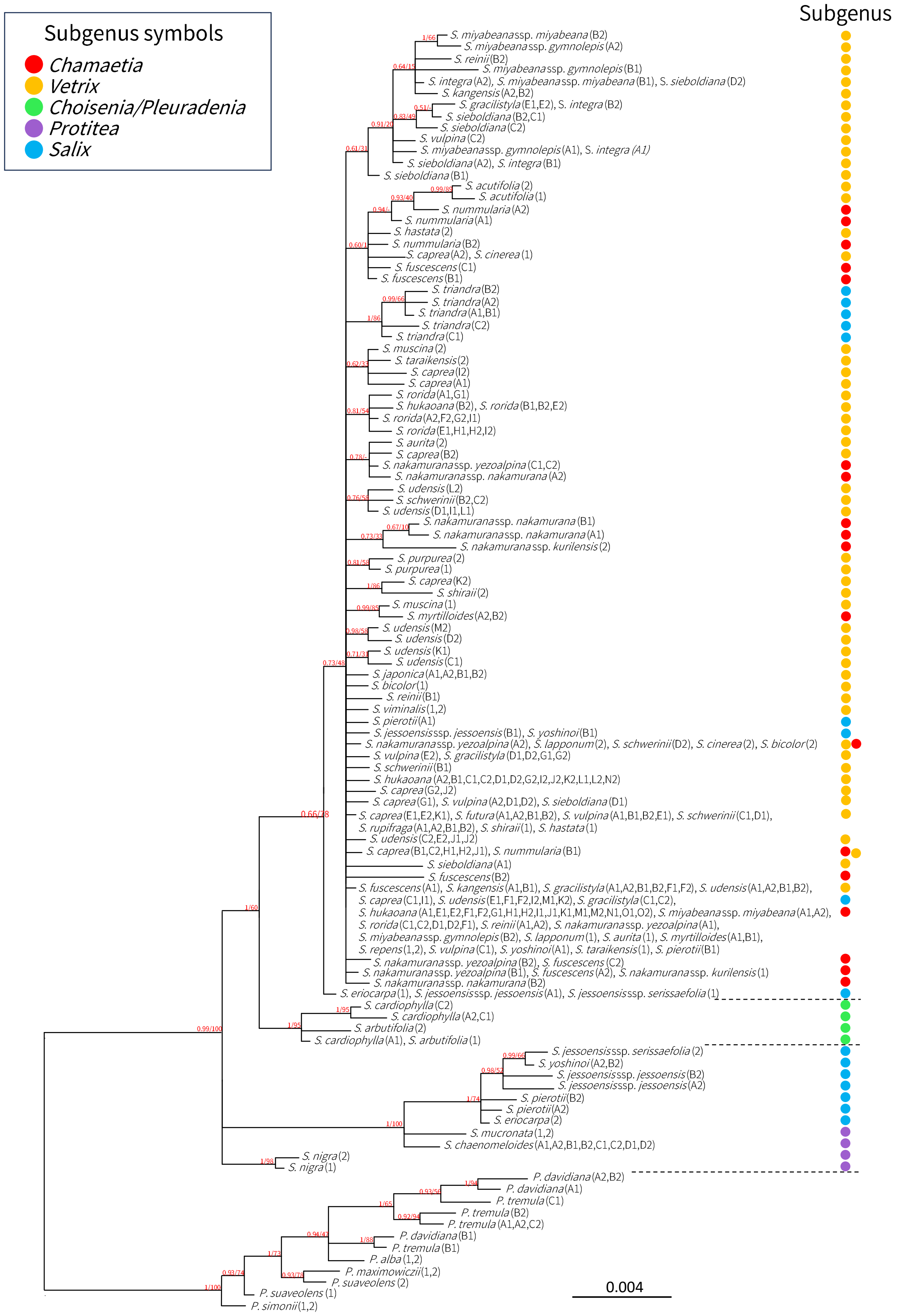

Subgenus symbols
Chamaetia
Vetrix
Choisenia/Pleuradenia
Protitea
Salix
Subgenus
S. miyabeana ssp. miyabeana (B2)
S. miyabeana ssp. gymnolepis (A2)
S. reinii (B2)
S. miyabeana ssp. gymnolepis (B1)
S. integra (A2), S. miyabeana ssp. miyabeana (B1), S. sieboldiana (D2)
S. kangensis (A2,B2)
S. gracilistyla (E1,E2), S. integra (B2)
S. sieboldiana (B2,C1)
S. sieboldiana (C2)
S. vulpina (C2)
S. miyabeana ssp. gymnolepis (A1), S. integra (A1)
S. sieboldiana (A2), S. integra (B1)
S. sieboldiana (B1)
S. acutifolia (2)
S. acutifolia (1)
S. nummularia (A2)
S. nummularia (A1)
S. hastata (2)
S. nummularia (B2)
S. caprea (A2), S. cinerea (1)
S. fuscescens (C1)
S. fuscescens (B1)
S. triandra (B2)
S. triandra (A2)
S. triandra (A1,B1)
S. triandra (C2)
S. triandra (C1)
S. muscina (2)
S. taraikensis (2)
S. caprea (I2)
S. caprea (A1)
S. rorida (A1,G1)
S. hukaoana (B2), S. rorida (B1,B2,E2)
S. rorida (A2,F2,G2,I1)
S. rorida (E1,H1,H2,I2)
S. aurita (2)
S. caprea (B2)
S. nakamurana ssp. yezoalpina (C1,C2)
S. nakamurana ssp. nakamurana (A2)
S. udensis (L2)
S. schwerinii (B2,C2)
S. udensis (D1,I1,L1)
S. nakamurana ssp. nakamurana (B1)
S. nakamurana ssp. nakamurana (A1)
S. nakamurana ssp. kurilensis (2)
S. purpurea (2)
S. purpurea (1)
S. caprea (K2)
S. shiraii (2)
S. muscina (1)
S. myrtilloides (A2,B2)
S. udensis (M2)
S. udensis (D2)
S. udensis (K1)
S. udensis (C1)
S. japonica (A1,A2,B1,B2)
S. bicolor (1)
S. reinii (B1)
S. viminalis (1,2)
S. pierotii (A1)
S. jessoensis ssp. jessoensis (B1), S. yoshinoi (B1)
S. nakamurana ssp. yezoalpina (A2), S. lapponum (2), S. schwerinii (D2), S. cinerea (2), S. bicolor (2)
S. vulpina (E2), S. gracilistyla (D1,D2,G1,G2)
S. schwerinii (B1)
S. hukaoana (A2,B1,C1,C2,D1,D2,G2,I2,J2,K2,L1,L2,N2)
S. caprea (G2,J2)
S. caprea (G1), S. vulpina (A2,D1,D2), S. sieboldiana (D1)
S. caprea (E1,E2,K1), S. futura (A1,A2,B1,B2), S. vulpina (A1,B1,B2,E1), S. schwerinii (C1,D1),
S. rupifraga (A1,A2,B1,B2), S. shiraii (1), S. hastata (1)
S. udensis (C2,E2,J1,J2)
S. caprea (B1,C2,H1,H2,J1), S. nummularia (B1)
S. sieboldiana (A1)
S. fuscescens (B2)
S. fuscescens (A1), S. kangensis (A1,B1), S. gracilistyla (A1,A2,B1,B2,F1,F2), S. udensis (A1,A2,B1,B2),
S. caprea (C1,I1), S. udensis (E1,F1,F2,I2,M1,K2), S. gracilistyla (C1,C2),
S. hukaoana (A1,E1,E2,F1,F2,G1,H1,H2,I1,J1,K1,M1,M2,N1,O1,O2), S. miyabeana ssp. miyabeana (A1,A2),
S. rorida (C1,C2,D1,D2,F1), S. reinii (A1,A2), S. nakamurana ssp. yezoalpina (A1),
S. miyabeana ssp. gymnolepis (B2), S. lapponum (1), S. aurita (1), S. myrtilloides (A1,B1),
S. repens (1,2), S. vulpina (C1), S. yoshinoi (A1), S. taraikensis (1), S. pierotii (B1)
S. nakamurana ssp. yezoalpina (B2), S. fuscescens (C2)
S. nakamurana ssp. yezoalpina (B1), S. fuscescens (A2), S. nakamurana ssp. kurilensis (1)
S. nakamurana ssp. nakamurana (B2)
S. eriocarpa (1), S. jessoensis ssp. jessoensis (A1), S. jessoensis ssp. serissaefolia (1)
S. cardiophylla (C2)
S. cardiophylla (A2,C1)
S. arbutifolia (2)
S. cardiophylla (A1), S. arbutifolia (1)
S. jessoensis ssp. serissaefolia (2)
S. yoshinoi (A2,B2)
S. jessoensis ssp. jessoensis (B2)
S. jessoensis ssp. jessoensis (A2)
S. pierotii (B2)
S. pierotii (A2)
S. eriocarpa (2)
S. mucronata (1,2)
S. chaenomeloides (A1,A2,B1,B2,C1,C2,D1,D2)
S. nigra (2)
S. nigra (1)
P. davidiana (A2,B2)
P. davidiana (A1)
P. tremula (C1)
P. tremula (B2)
P. tremula (A1,A2,C2)
P. davidiana (B1)
P. tremula (B1)
P. alba (1,2)
P. maximowiczii (1,2)
P. suaveolens (2)
P. suaveolens (1)
P. simonii (1,2)
1/66
0.64/15
0.51/-
0.83/49
0.91/20
0.61/31
0.99/89
0.93/40
0.94/-
0.60/1
0.99/66
1/86
0.62/33
0.81/54
0.78/-
0.76/58
0.67/16
0.73/33
0.81/58
1/86
0.99/85
0.98/58
0.71/31
0.73/48
0.66/28
1/60
1/95
1/95
0.99/100
0.99/66
0.98/52
1/74
1/100
1/98
1/94
0.93/56
1/65
0.92/94
0.94/47
1/88
1/73
0.93/74
0.93/78
1/100
0.004

b ***PGI***

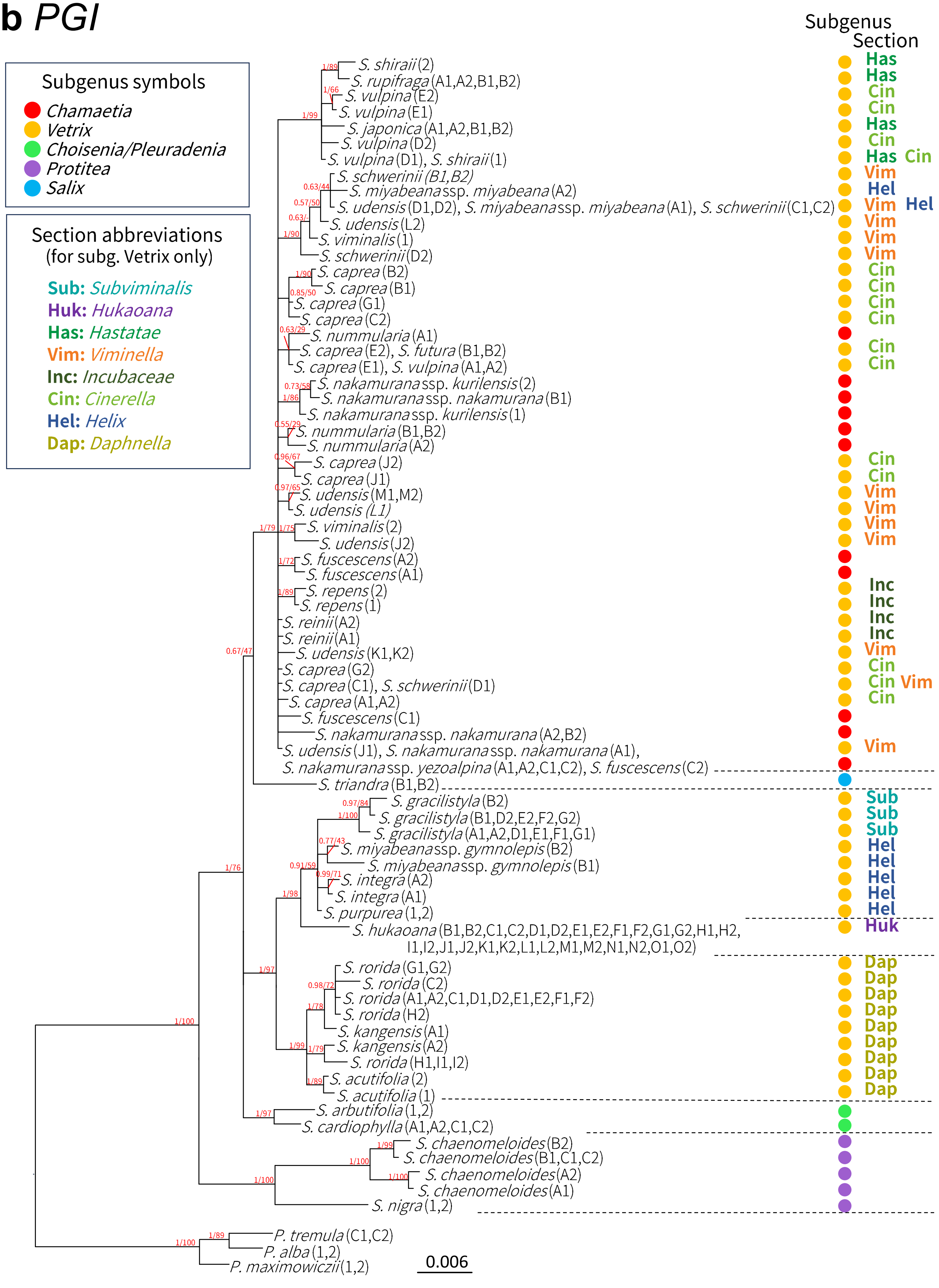
Subgenus
Section
Subgenus symbols
Chamaetia
Vetrix
Choisenia/Pleuradenia
Protitea
Salix
Section abbreviations
(for subg. Vetrix only)
Sub: Subviminalis
Huk: Hukaoana
Has: Hastatae
Vim: Viminella
Inc: Incubaceae
Cin: Cinerella
Hel: Helix
Dap: Daphnella
S. shiraii (2)
S. rupifraga (A1,A2,B1,B2)
S. vulpina (E2)
S. vulpina (E1)
S. japonica (A1,A2,B1,B2)
S. vulpina (D2)
S. vulpina (D1), S. shiraii (1)
S. schwerinii (B1,B2)
S. miyabeana ssp. miyabeana (A2)
S. udensis (D1,D2), S. miyabeana ssp. miyabeana (A1), S. schwerinii (C1,C2)
S. udensis (L2)
S. viminalis (1)
S. schwerinii (D2)
S. caprea (B2)
S. caprea (B1)
S. caprea (G1)
S. caprea (C2)
S. nummularia (A1)
S. caprea (E2), S. futura (B1,B2)
S. caprea (E1), S. vulpina (A1,A2)
S. nakamurana ssp. kurilensis (2)
S. nakamurana ssp. nakamurana (B1)
S. nakamurana ssp. kurilensis (1)
S. nummularia (B1,B2)
S. nummularia (A2)
S. caprea (J2)
S. caprea (J1)
S. udensis (M1,M2)
S. udensis (L1)
S. viminalis (2)
S. udensis (J2)
S. fuscescens (A2)
S. fuscescens (A1)
S. repens (2)
S. repens (1)
S. reinii (A2)
S. reinii (A1)
S. udensis (K1,K2)
S. caprea (G2)
S. caprea (C1), S. schwerinii (D1)
S. caprea (A1,A2)
S. fuscescens (C1)
S. nakamurana ssp. nakamurana (A2,B2)
S. udensis (J1), S. nakamurana ssp. nakamurana (A1),
S. nakamurana ssp. yezoalpina (A1,A2,C1,C2), S. fuscescens (C2)
S. triandra (B1,B2)
S. gracilistyla (B2)
S. gracilistyla (B1,D2,E2,F2,G2)
S. gracilistyla (A1,A2,D1,E1,F1,G1)
S. miyabeana ssp. gymnolepis (B2)
S. miyabeana ssp. gymnolepis (B1)
S. integra (A2)
S. integra (A1)
S. purpurea (1,2)
S. hukaoana (B1,B2,C1,C2,D1,D2,E1,E2,F1,F2,G1,G2,H1,H2,
I1,I2,J1,J2,K1,K2,L1,L2,M1,M2,N1,N2,O1,O2)
S. rorida (G1,G2)
S. rorida (C2)
S. rorida (A1,A2,C1,D1,D2,E1,E2,F1,F2)
S. rorida (H2)
S. kangensis (A1)
S. kangensis (A2)
S. rorida (H1,I1,I2)
S. acutifolia (2)
S. acutifolia (1)
S. arbutifolia (1,2)
S. cardiophylla (A1,A2,C1,C2)
S. chaenomeloides (B2)
S. chaenomeloides (B1,C1,C2)
S. chaenomeloides (A2)
S. chaenomeloides (A1)
S. nigra (1,2)
P. tremula (C1,C2)
P. alba (1,2)
P. maximowiczii (1,2)
0.006
1/89
1/66
1/99
0.63/44
0.57/50
0.63/-
1/90
1/90
0.85/50
0.63/29
0.73/58
1/86
0.55/29
0.96/67
0.97/65
1/79
1/75
1/72
1/89
0.67/47
0.97/84
1/100
0.77/43
0.91/59
0.99/71
1/98
1/76
1/97
0.98/72
1/78
1/99
1/79
1/89
1/100
1/97
1/99
1/100
1/100
1/100
1/89
1/100
Has
Has
Cin
Cin
Has
Cin
Has Cin
Vim
Hel
Vim Hel
Vim
Vim
Vim
Cin
Cin
Cin
Cin
Cin
Cin
Cin
Cin
Vim
Vim
Vim
Vim
Inc
Inc
Inc
Inc
Vim
Cin
Cin Vim
Cin
Vim
Sub
Sub
Sub
Hel
Hel
Hel
Hel
Hel
Huk
Dap
Dap
Dap
Dap
Dap
Dap
Dap
Dap
Dap

c *ncpGS*

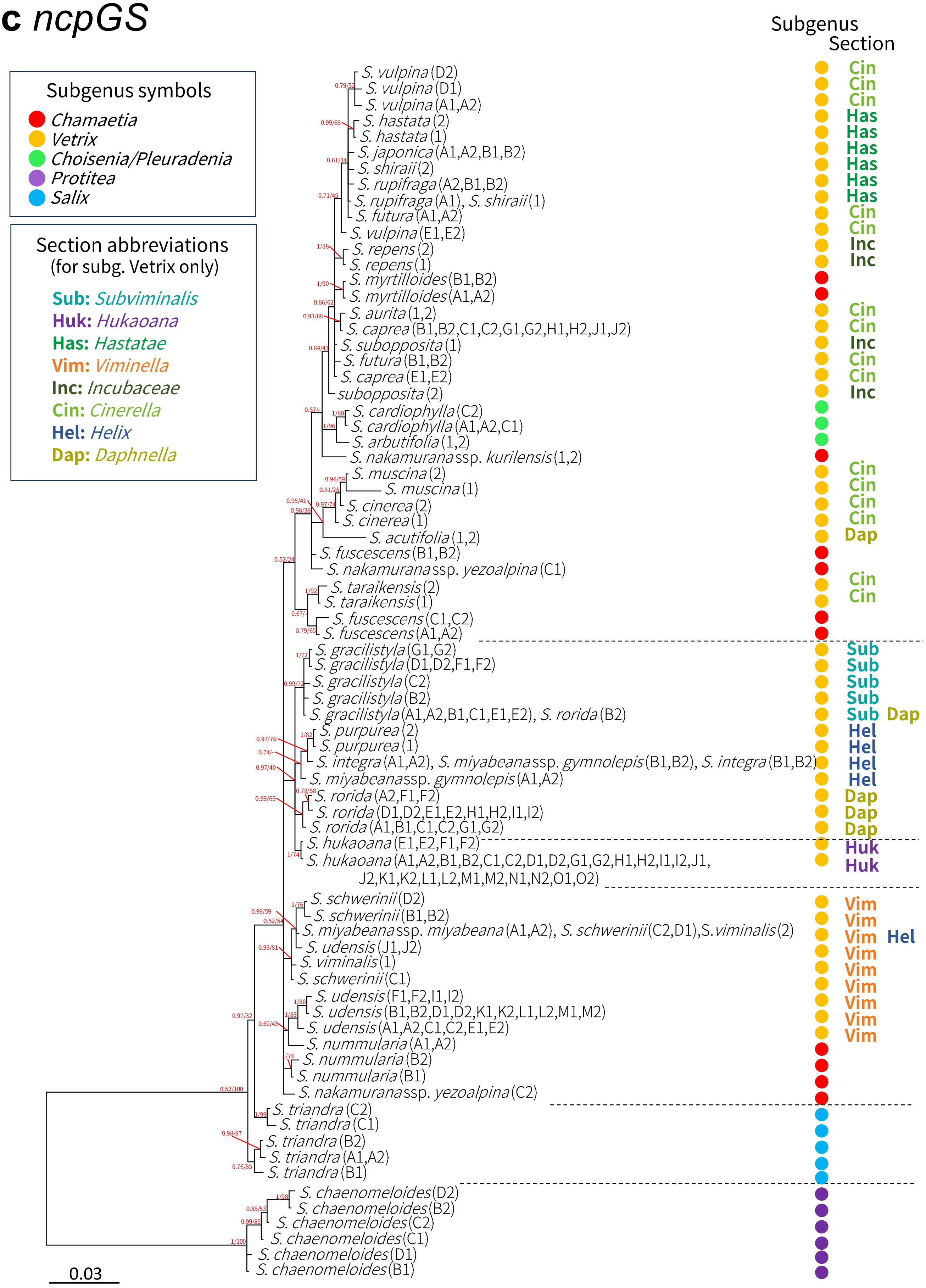
Subgenus
Section
Subgenus symbols
Chamaetia
Vetrix
Choisenia/Pleuradenia
Protitea
Salix
Section abbreviations
(for subg. Vetrix only)
Sub: Subviminalis
Huk: Hukaoana
Has: Hastatae
Vim: Viminella
Inc: Incubaceae
Cin: Cinerella
Hel: Helix
Dap: Daphnella
S. vulpina (D2) Cin
S. vulpina (D1) Cin
S. vulpina (A1,A2) Cin
S. hastata (2) Has
S. hastata (1) Has
S. japonica (A1,A2,B1,B2) Has
S. shiraii (2) Has
S. rupifraga (A2,B1,B2) Has
S. rupifraga (A1), S. shiraii (1) Has
S. futura (A1,A2) Cin
S. vulpina (E1,E2) Cin
S. repens (2) Inc
S. repens (1) Inc
S. myrtilloides (B1,B2)
S. myrtilloides (A1,A2)
S. aurita (1,2) Cin
S. caprea (B1,B2,C1,C2,G1,G2,H1,H2,J1,J2) Cin
S. subopposita (1) Inc
S. futura (B1,B2) Cin
S. caprea (E1,E2) Cin
subopposita (2) Inc
S. cardiophylla (C2)
S. cardiophylla (A1,A2,C1)
S. arbutifolia (1,2)
S. nakamurana ssp. kurilensis (1,2)
S. muscina (2) Cin
S. muscina (1) Cin
S. cinerea (2) Cin
S. cinerea (1) Cin
S. acutifolia (1,2) Dap
S. fuscescens (B1,B2)
S. nakamurana ssp. yezoalpina (C1)
S. taraikensis (2) Cin
S. taraikensis (1) Cin
S. fuscescens (C1,C2)
S. fuscescens (A1,A2)
S. gracilistyla (G1,G2) Sub
S. gracilistyla (D1,D2,F1,F2) Sub
S. gracilistyla (C2) Sub
S. gracilistyla (B2) Sub
S. gracilistyla (A1,A2,B1,C1,E1,E2), S. rorida (B2) Sub Dap
S. purpurea (2) Hel
S. purpurea (1) Hel
S. integra (A1,A2), S. miyabeana ssp. gymnolepis (B1,B2), S. integra (B1,B2) Hel
S. miyabeana ssp. gymnolepis (A1,A2) Hel
S. rorida (A2,F1,F2) Dap
S. rorida (D1,D2,E1,E2,H1,H2,I1,I2) Dap
S. rorida (A1,B1,C1,C2,G1,G2) Dap
S. hukaoana (E1,E2,F1,F2) Huk
S. hukaoana (A1,A2,B1,B2,C1,C2,D1,D2,G1,G2,H1,H2,I1,I2,J1,
J2,K1,K2,L1,L2,M1,M2,N1,N2,O1,O2) Huk
S. schwerinii (D2) Vim
S. schwerinii (B1,B2) Vim
S. miyabeana ssp. miyabeana (A1,A2), S. schwerinii (C2,D1),S. viminalis (2) Vim Hel
S. udensis (J1,J2) Vim
S. viminalis (1) Vim
S. schwerinii (C1) Vim
S. udensis (F1,F2,I1,I2) Vim
S. udensis (B1,B2,D1,D2,K1,K2,L1,L2,M1,M2) Vim
S. udensis (A1,A2,C1,C2,E1,E2) Vim
S. nummularia (A1,A2)
S. nummularia (B2)
S. nummularia (B1)
S. nakamurana ssp. yezoalpina (C2)
S. triandra (C2)
S. triandra (C1)
S. triandra (B2)
S. triandra (A1,A2)
S. triandra (B1)
S. chaenomeloides (D2)
S. chaenomeloides (B2)
S. chaenomeloides (C2)
S. chaenomeloides (C1)
S. chaenomeloides (D1)
S. chaenomeloides (B1)
0.03
