# Supplementary material for: Preliminary phylogenetic insights into Japanese willows (Salix) using low-copy nuclear genes, with emphasis on endemic species
Source: J Plant Res. 2026 Jun 15;139(4):575–91. doi: 10.1007/s10265-026-01728-x (PMC13332978; doi:10.1007/s10265-026-01728-x)
Supplement: Supplementary file 3 — Supplementary Material 3 [file 10265_2026_1728_MOESM3_ESM.pdf]

Title: Preliminary phylogenetic insights into Japanese willows (*Salix*) using low-copy nuclear genes, with emphasis on endemic species

Journal: Journal of Plant Research

Authors: Satoshi Kikuchi, Suzuki Setsuko, Teruyoshi Nagamitsu, Wajiro Suzuki
Affiliation: Hokkaido Research Center, Forestry and Forest Products Research Institute, Japan
Corresponding author: Satoshi Kikuchi
Email: kikuchi_satoshi450@ffpri.go.jp

Online Resource 3

The NeighborNet network of *Salix*, based on (**a**) nuclear *ncpGS* and (**b**) *PGI* sequences, showing the possibility of hybrid formation in *S. nakamurana* subsp. *nakamurana* and *S. miyabeana* subsp. *miyabeana* (shown in boxes). The network was constructed using uncorrected P distances based on concatenated chloroplast sequences and three nuclear genes. Splits with bootstrap support >50% and >80% were shown using increasingly thicker line weights.

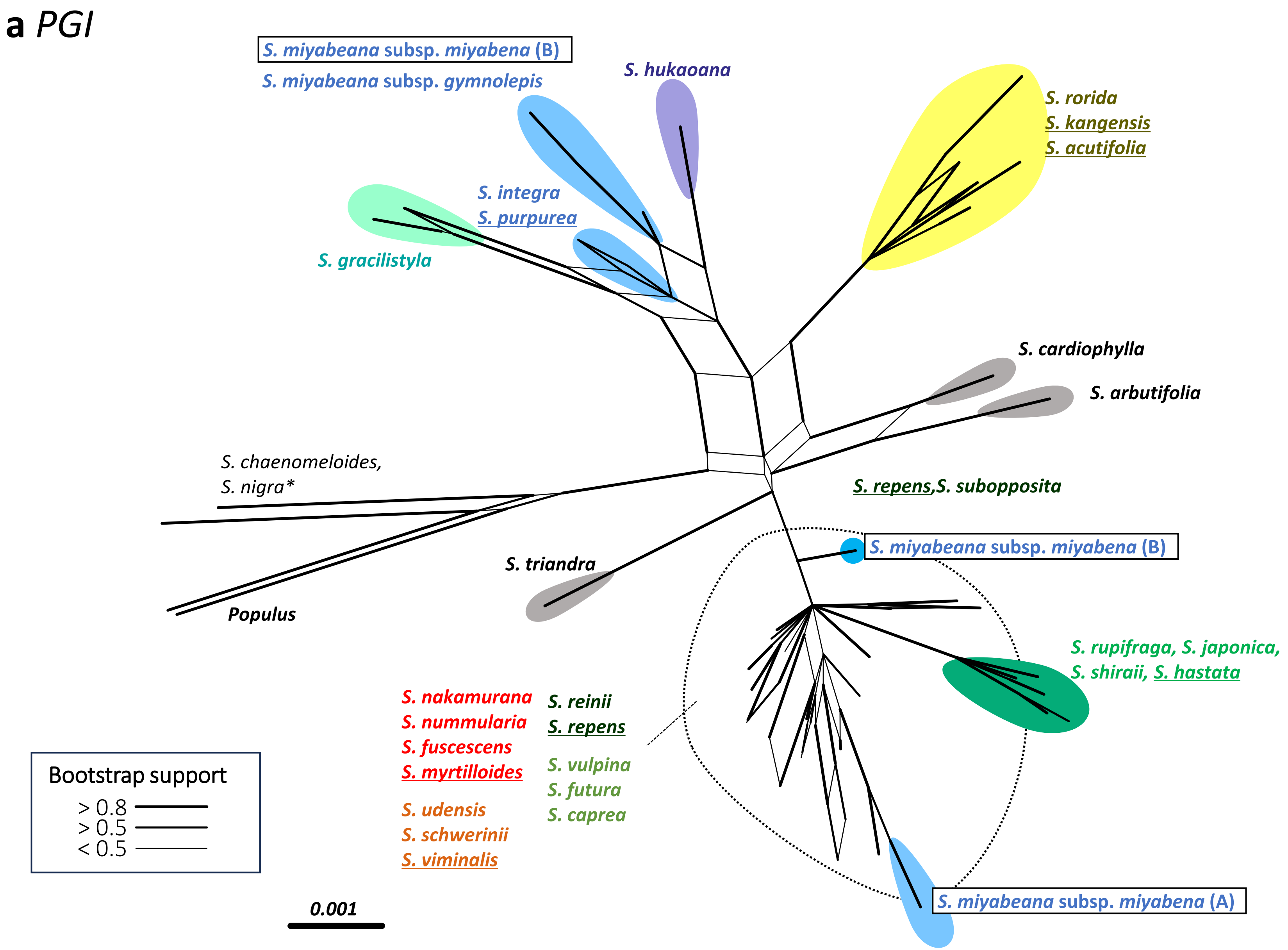
a PGI
S. miyabeana subsp. miyabena (B)
S. miyabeana subsp. gymnolepis
S. hukaoana
S. rorida
S. kangensis
S. acutifolia
S. integra
S. purpurea
S. gracilistyla
S. cardiophylla
S. arbutifolia
S. chaenomeloides,
S. nigra*
S. repens,S. subopposita
S. miyabeana subsp. miyabena (B)
S. triandra
Populus
S. rupifraga, S. japonica,
S. shiraii, S. hastata
S. nakamurana
S. nummularia
S. fuscescens
S. myrtilloides
S. udensis
S. schwerinii
S. viminalis
S. reinii
S. repens
S. vulpina
S. futura
S. caprea
Bootstrap support
> 0.8
> 0.5
< 0.5
0.001
S. miyabeana subsp. miyabena (A)

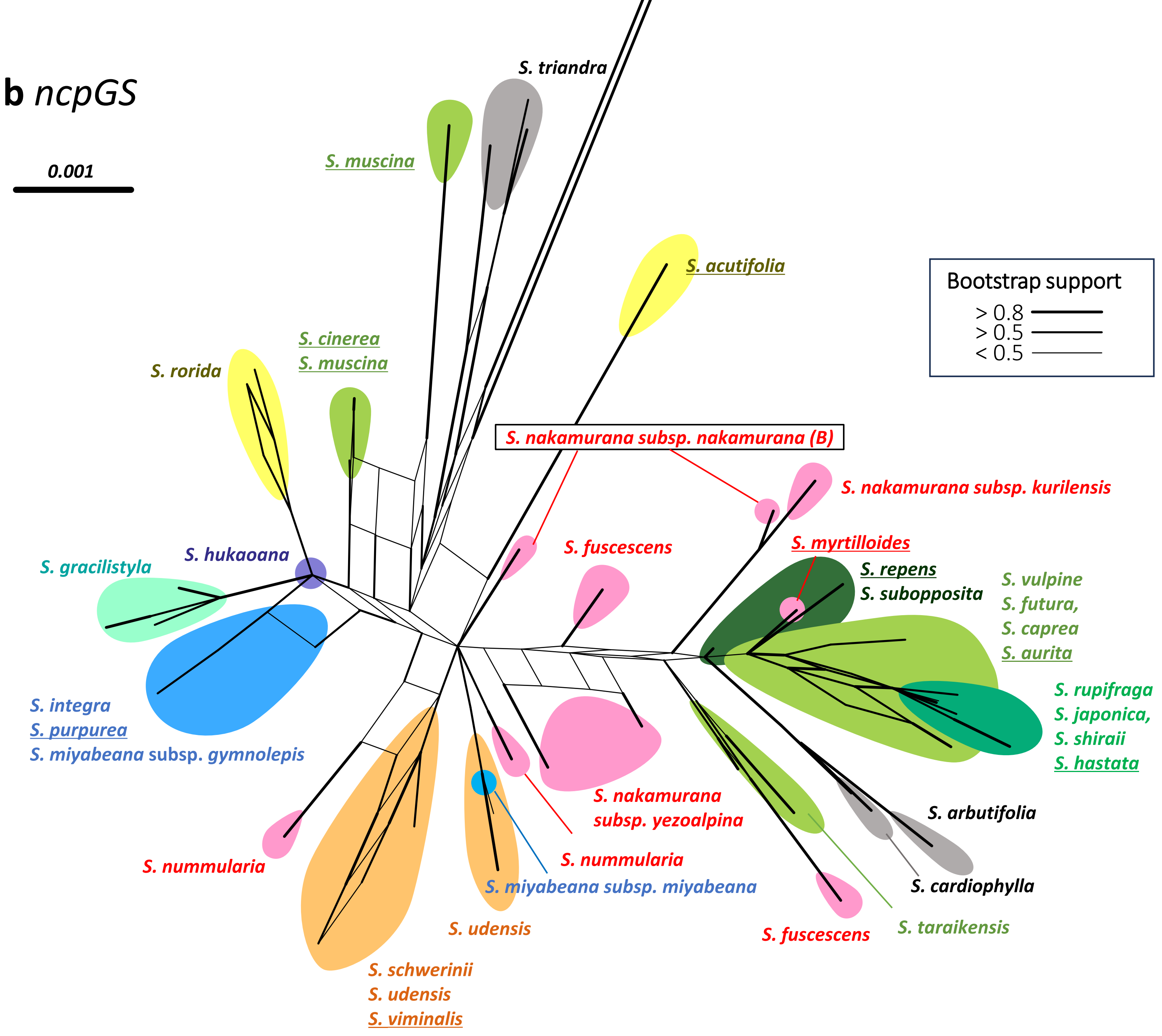

b ncpGS
0.001
S. triandra
S. muscina
S. acutifolia
Bootstrap support
> 0.8
> 0.5
< 0.5
S. cinerea
S. muscina
S. rorida
S. nakamurana subsp. nakamurana (B)
S. nakamurana subsp. kurilensis
S. hukaoana
S. gracilistyla
S. fuscescens
S. myrtilloides
S. repens
S. subopposita
S. vulpine
S. futura,
S. caprea
S. aurita
S. rupifraga
S. japonica,
S. shiraii
S. hastata
S. integra
S. purpurea
S. miyabeana subsp. gymnolepis
S. arbutifolia
S. nakamurana
subsp. yezoalpina
S. nummularia
S. nummularia
S. miyabeana subsp. miyabeana
S. cardiophylla
S. udensis
S. fuscescens
S. taraikensis
S. schwerinii
S. udensis
S. viminalis
