# Supplementary material for: Preliminary phylogenetic insights into Japanese willows (Salix) using low-copy nuclear genes, with emphasis on endemic species
Source: J Plant Res. 2026 Jun 15;139(4):575–91. doi: 10.1007/s10265-026-01728-x (PMC13332978; doi:10.1007/s10265-026-01728-x)
Supplement: Supplementary file 4 — Supplementary Material 4 [file 10265_2026_1728_MOESM4_ESM.pdf]

Title: Preliminary phylogenetic insights into Japanese willows (*Salix*) using low-copy nuclear genes, with emphasis on endemic species

Journal: Journal of Plant Research

Authors: Satoshi Kikuchi, Suzuki Setsuko, Teruyoshi Nagamitsu, Wajiro Suzuki
Affiliation: Hokkaido Research Center, Forestry and Forest Products Research Institute, Japan
Corresponding author: Satoshi Kikuchi
Email: kikuchi_satoshi450@ffpri.go.jp

Online Resource 4

Ambiguous nucleotide sites of *PGI* and *ncpGS* genes in the putative hybrid individual (*S. miyabeana* subsp. *miyabeana* and *S. nakamurana* subsp. *nakamurana,* respectively). Ambiguous bases are shown using the standard IUPAC ambiguity codes (M = A/C, R = A/G, Y = C/T, S = C/G, K = T/G, W = A/T). For comparison, the nucleotide states of the potential parental species are listed.

PGI

Position (bps)	140	238	274	277	281	391	476	750	864	874	877	896	902	916
***S. miyabeana* ssp. *miyabeana* B**	**W**	**Y**	**Y**	**W**	**Y**	**Y**	**W**	**R**	**R**	**Y**	**Y**	**K**	**M**	**W**
S. miyabeana ssp. *gymnolepis* B	T	Y	T	A	C	T	T	A	G	Y	T	G	C	W
S. udensis L	A	T	C	T	T	T	A	G	T	T	C	T	A	A
S. caprea C,D	A	T	C	T	T	T	A	G	T	T	C	T	A	A
S. schwerinii C	A	T	C	T	T	T	A	G	T	T	C	T	A	A
S. viminalis	A	T	C	T	T	T	A	G	T	T	C	T	A	A
S. nakamurana B	A	T	C	T	T	T	A	G	T	T	C	T	A	A
S. yezoalpina A	A	T	C	T	T	T	A	G	T	T	C	T	A	A
S. reinii A	A	T	C	T	T	T	A	G	T	T	C	T	A	A

ncpGS

Position (bps)	22	32	52	220	235	260	267	302	303	309	345	408	481
***S. nakamurana* ssp. *nakamurana* B**	**Y**	**Y**	**K**	**Y**	**Y**	**Y**	**R**	**S**	**S**	**M**	**Y**	**K**	**R**
S. nakamurana ssp. *kurilensis* B	T	T	T	C	C	C	A	C	C	A	C	T	A
S. nakamurana ssp. *yezoalpina* C	Y	C	G	T	T	T	R	G	G	C	C	G	R
