# Supplementary material for: Preliminary phylogenetic insights into Japanese willows (Salix) using low-copy nuclear genes, with emphasis on endemic species
Source: J Plant Res. 2026 Jun 15;139(4):575–91. doi: 10.1007/s10265-026-01728-x (PMC13332978; doi:10.1007/s10265-026-01728-x)
Supplement: Supplementary file 5 — Supplementary Material 5 [file 10265_2026_1728_MOESM5_ESM.docx]

**Captions for Online Resources**

**Online Resource 1**:

List of samples (i.e., species, isolates, and locations) and GenBank accession numbers for sequenced genes.

**Online Resource 2**

Phylogenetic trees (gene trees) based on the phased sequences of (**a**) nuclear 6*PG*, (**b**) *PGI*, and (**c**) *ncpGS* genes as reconstructed using the Bayesian inference (BI) method. Sequences were collapsed into unique haplotypes using DnaSP to form reduced datasets. The numbers above the branches represent Bayesian posterior probability and MP bootstrap values. “–“ indicates that the node was not supported in MP analysis.

**Online Resource 3**

The NeighborNet network of *Salix*, based on (**a**) nuclear *ncpGS* and (**b**) *PGI* sequences, showing the possibility of hybrid formation in *S. nakamurana* subsp. *nakamurana* and *S. miyabeana* subsp. *miyabeana* (shown in boxes). The network was constructed using uncorrected P distances based on concatenated chloroplast sequences and three nuclear genes. Splits with bootstrap support >50% and >80% were shown using increasingly thicker line weights.

**Online Resource 4**

Ambiguous nucleotide sites of *PGI* and *ncpGS* genes in the putative hybrid individual (*S. miyabeana* subsp. *miyabeana* and *S. nakamurana* subsp. *nakamurana*, respectively). Ambiguous bases are shown using the standard IUPAC ambiguity codes (M = A/C, R = A/G, Y = C/T, S = C/G, K = T/G, W = A/T). For comparison, the nucleotide states of the potential parental species are listed.
